# Utility of serum amyloid A as a potential prognostic biomarker of aneurysmal subarachnoid hemorrhage

**DOI:** 10.3389/fneur.2022.1099391

**Published:** 2023-01-12

**Authors:** Zhongbo Sun, Yaqiang Li, Fu Chang, Ke Jiang

**Affiliations:** ^1^Department of Neurosurgery, First Affiliated Hospital of Anhui University of Science and Technology (First People's Hospital of Huainan), Huainan, China; ^2^Department of Neurology, People's Hospital of Lixin County, Bozhou, China

**Keywords:** inflammation, aneurysmal subarachnoid hemorrhage, aSAH, serum amyloid A, SAA

## Abstract

**Objectives:**

Inflammation plays a vital role in the aneurysmal subarachnoid hemorrhage (aSAH), while serum amyloid A (SAA) has been identified as an inflammatory biomarker. The present study aimed to elucidate the relationship between SAA concentrations and prognosis in aSAH.

**Methods:**

From prospective analyses of patients admitted to our department between March 2016 and August 2022, aSAH patients with complete medical records were evaluated. Meanwhile, the healthy control group consisted of the age and sex matched individuals who came to our hospital for healthy examination between March 2018 and August 2022. SAA level was measured by enzyme-linked immunosorbent assay kit (Invitrogen Corp). The Glasgow Outcome Scale (GOS) was used to classify patients into good (GOS score of 4 or 5) and poor (GOS score of 1, 2, or 3) outcome.

**Results:**

456 patients were enrolled in the study, thereinto, 200 (43.86%) patients had a poor prognosis at the 3-months follow-up. Indeed, the SAA of poor outcome group were significantly increased compared to good outcome group and healthy control group [36.44 (32.23–41.00) vs. 28.99 (14.67–34.12) and 5.64 (3.43–7.45), *P* < 0.001]. In multivariate analyses, SAA served for independently predicting the poor outcome after aICH at 3 months [OR:1.129 (95% CI, 1.081–1.177), *P* < 0.001]. After adjusting the underlying confounding factors, the odds ratio (OR) of depression after aSAH was 2.247 (95% CI: 1.095–4.604, *P* = 0.021) for the highest tertile of SAA relative to the lowest tertile. With an AUC of 0.807 (95% CI, 0.623–0.747), SAA demonstrated an obviously better discriminatory ability relative to CRP, WBC, and IL-6. SAA as an indicator for predicting poor outcome after aSAH had an optimal cut-off value of 30.28, and the sensitivity and specificity were 61.9 and 78.7%, respectively.

**Conclusions:**

Elevated level of SAA was associated with poor outcome at 3 months, suggesting that SAA might be a useful inflammatory markers to predict prognosis after aSAH.

## 1. Introduction

Aneurysmal subarachnoid hemorrhage (aSAH) is a serious life-threatening acute cerebrovascular disease that severely damages the patient's central nervous system and has pathophysiological effects on several organs of the body (1, 2). The mortality rate of aSAH patients is reported to be about 33%, and at least 20% of survivors have poor long-term functional outcomes. Additionally, the 3-month mortality rate of aSAH patients is as high as 47–49%, and most of the survivors still have serious sequelae, which seriously affect the quality of survival (3, 4). Clinically, Hunt and Hess (HH) scale and Modified Fisher (MF) score are mostly used to predict the prognosis of aSAH at an early stage, but this approach is highly subjective and there are cases in which the HH and MF score are high at the time of admission, but the prognosis is good. Therefore, relying on only these approaches to estimate the prognosis of patients with aSAH remains limited.

Although the pathophysiological mechanisms of early brain injury after aSAH are complex, inflammation has been shown to develop in early stages sufficient to be involved in early brain injury after aSAH (5–7). Accumulating studies have shown that inflammatory biomarkers have drawn the attention of clinicians and can be used to assess the severity of aSAH and reflect prognosis (3, 8, 9). Serum amyloid A (SAA) is a apolipoprotein that is presented in the high-density lipoprotein (HDL) fraction of serum and is responsible for the chemotactic recruitment of inflammatory cells to sites of inflammation (10). It is also mainly synthesized by hepatocytes and plays a very important role in the diagnosis of acute and chronic inflammation as a widely used non-specific inflammatory marker in clinical practice. Notably, the concentration of SAA was significantly increased in the blood and liver of traumatic brain injury mice in the early stages (11). Elevated SAA concentrations in peripheral blood have been reported in adult patients suffering from traumatic brain injury and severe polytrauma (12). In addition, a recent study revealed that elevated serum SAA concentrations, which were strongly associated with inflammation and hemorrhagic severity, were independently associated with mortality and poor outcomes after intracerebral hemorrhage (ICH) (13). Other studies have found that an increased SAA is significantly correlated with inflammatory conditions, including atherosclerosis, post-stroke cognitive impairment, and myocardial infarction (14–16).

By now, few studies have focused on SAA as a prognostic marker of neurological outcome, and no study has suggested a cutoff SAA for predicting poor outcome in patients with aSAH. Therefore, the study aimed at investigating the relationship between SAA and poor outcome in patients with aSAH, as well as further establishing a cutoff SAA value as a prognostic marker for assessing functional outcome in aSAH patients.

## 2. Materials and methods

### 2.1. Subjects

**Figure 1:**
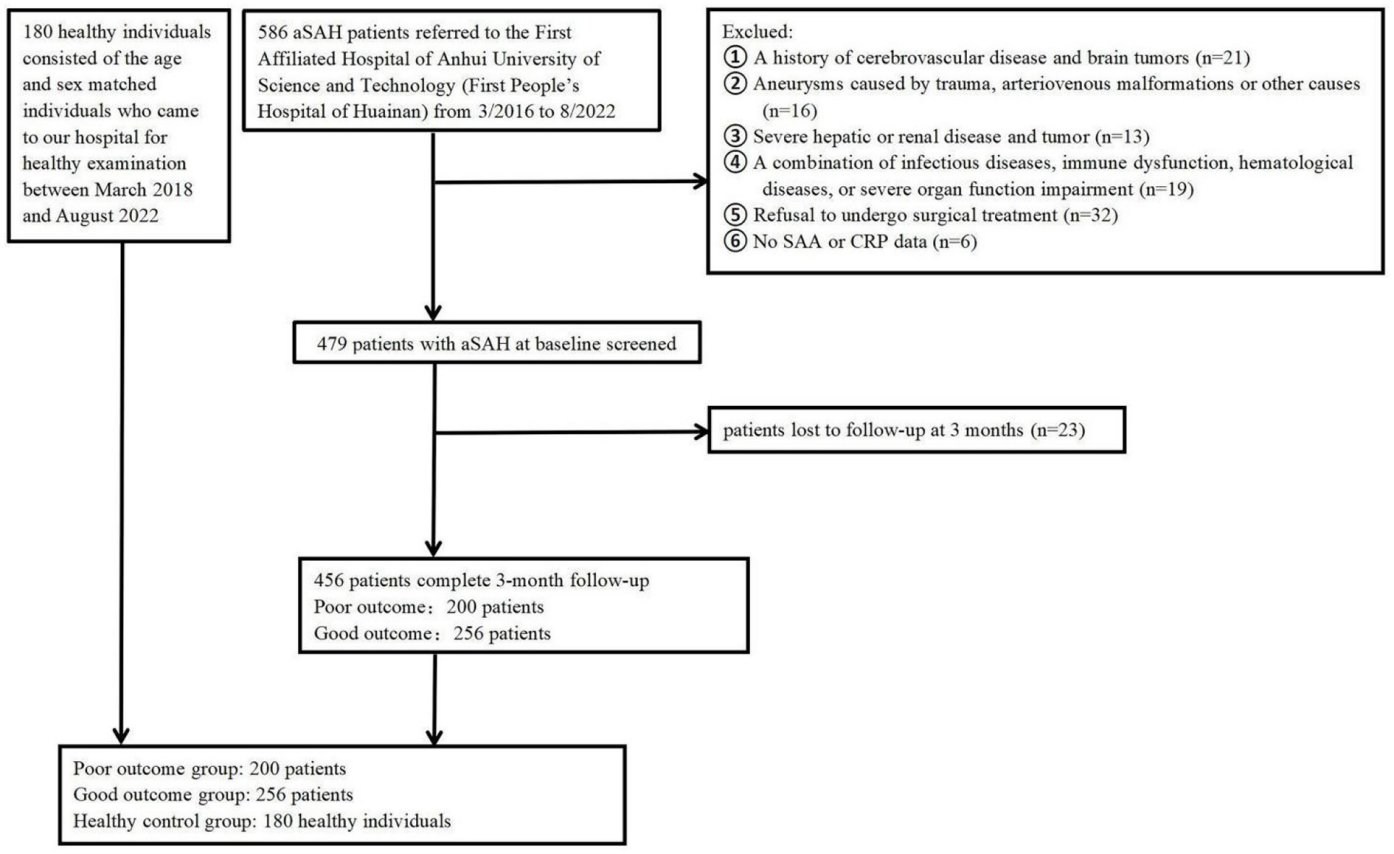
Flowchart of participant selection. aSAH, Aneurysmal subarachnoid hemorrhage; SAA, serum amyloid A; CRP, C-reactive protein.

The present analysis was conducted as a component of a larger study that aimed to investigate clinical outcome in aneurysmal subarachnoid hemorrhage (aSAH) using a 3-month naturalistic prospective design. Patients with aSAH were consecutively enrolled from the First Affiliated Hospital of Anhui University of Science and Technology (First People's Hospital of Huainan) from March 2016 to August 2022, within 24 h after the onset of aSAH. Patients diagnosed with aSAH through the following were included: subarachnoid hemorrhage diagnosed by head computed tomography, and an aneurysm confirmed with digital subtraction angiography (DSA) or computed tomography angiography (CTA). Patients were not included if they: (1) had aneurysms caused by trauma, arteriovenous malformations or other causes; (2) had no SAA and CRP data. (3) had a history of cerebrovascular disease and brain tumors. (4) had a combination of infectious diseases, immune dysfunction, hematological diseases, or severe organ function impairment. (5) had refused to undergo surgical treatment. (6) had severe renal or liver diseases. At last, the study included 456 cases with aSAH in total (Figure 1). This study has obtained the approval of the institutional ethics review board of the participating center, and obtained all participants' written informed consent following the Helsinki Declaration of 1975. Meanwhile, the healthy control group consisted of the age and sex matched individuals who came to our hospital for healthy examination between March 2018 and August 2022. Controls (180 healthy individuals) were composed of 90 males and 90 females, as well as their mean value was 66.19 y (range, 35–75 y; SD, 8.43 y) at age.

All patients were treated according to guidelines for the management of aSAH, including prevention and management of rebleeding, vasospasm, and other complications (17). Determining the treatment of aneurysm, in the judgment of an experienced neurosurgeon, should be a comprehensive decision based on the characteristics of the patient and the aneurysm. All patients undergo oral or intravenous pumping of nimodipine to prevent vasospasm. Intracranial hypertension was treated with head elevation, mannitol injection and cerebrospinal fluid drainage, such as extraventricular or lumbar drainage. Antibiotherapy was only initiated when systemic clinical and biological criteria indicate the presence of a bacterial infection, for example, pneumonia, urinary tract infection, or bloodstream infection. Bacteriological sampling was performed at this time point and treatment was adjusted once results were obtained.

### 2.2. Data collection and assessment

We collected the sociodemographic and clinical characteristics regarding aSAH patients on admission, such as the age, gender, marital status, vascular risk factor (hypertension, diabetes mellitus, current smoking, alcohol consumption), diameter and location of aneurysm, operation of aneurysm (clip and coil), and adopted a standard case report form. The Hunt and Hess (HH) grade and World Federation of Neurosurgical Societies Scale served for assessing the clinical severity for all aSAH patients at the time of emergency department admission. Non-severe SAH is classified as HH grade I-III, and severe SAH is qualified as HH grade IV or V. The World Federation of Neurosurgical Societies Scale (WFNS 1–5) was used to classify patients into good (WFNS 1–2) and poor (WFNS 3–5) clinical status. The severity of the hemorrhage was determined using the modified fisher score. The primary outcome of interest was prognostic within 3 months following emergency department admission. Patients' clinical outcomes were determined by telephone interviews or clinical visits after 3 months of hemorrhage, using the Glasgow Outcome Scale (GOS). The GOS was used to dichotomize the patients in good (GOS score of 4 or 5) and poor (GOS score of 1, 2, or 3) outcome.

### 2.3. Blood collection and laboratory test

Blood samples were collected from peripheral veins within 30 min of arrival at the emergency department. The routine laboratory methods were conducted to measure serum glucose (G), hemoglobin (Hb), white blood cell count (WBC), platelet (Plt) count, C-reactive protein (CRP), total cholesterol (TC), triglycerides (TG), high density lipoprotein cholesterol (HDL-C), low density lipoprotein-cholesterol (LDL-C), apolipoprotein A (ApoA), and apolipoprotein B (ApoB). A sandwich enzyme-linked immunosorbent assay kit (BMS213-2, Invitrogen Corp, USA) served for measuring the IL-6. The SAA level was measured by enzyme-linked immunosorbent assay kit (EHSAA1, Invitrogen Corp, USA) and the operation was carried out strictly in accordance with the manufacturer's instructions. For the purpose of minimizing assay variation, professional clinical technicians who did not know the clinical outcomes or neuroimaging findings took charge of analyzing all samples in duplicate on the same day randomly.

### 2.4. Statistical analysis

SPSS 23.0 statistical software SPSS (Inc., Chicago, IL, USA) served for the statistical analyses. Data for continuous variables were in the form of mean ± standard deviation (SD) or median (interquartile range), which was decided by whether the tested data presented normal or non-normal distribution. The Kolmogorovor Smirnov test or Shapiro-Wilk test was used to investigate the normality of the data distribution. Chi-square assisted in evaluating categorical variables. In the case of conducting abnormal distribution test on the continuous variables, the Kruskal-Wallis test and Mann-Whitney U test served for comparing the difference among three groups and that between two groups, respectively. Student's *t*-test or one-way analysis of variance (ANOVA) served for analyzing the normally distributed continuous variables. We included the variables with *p* < 0.5 confirmed by the univariate analysis into the final multivariable analysis. The relationship between serum SAA concentrations and poor clinical outcome was assessed with a binary logistic regression analysis. Bivariate correlations were analyzed with Pearson or Spearman rank correlation analyses. The admission SAA was taken into account for dividing patients into tertiles (Q1 ≤ 29.23, Q2 29.33–35.44, Q3 ≥ 35.54). We applied three models for the multivariable regression analyses, for recognizing the factors that could predict the poor clinical outcome, with model 1 targeting age and sex; model 2 targeting model 1 as well as vascular risk factors; model 3 targeting variables with *P* < 0.05 confirmed by the univariate analysis (WBC, hunt and hess grade, modified fisher scale, WFNS score on admission, CRP and IL-6). Also, the association was in the form of OR with 95% CI. Besides, a ROC curve analysis assisted in identifying the cutoff point on the SAA levels on admission, which could the most sensitively and specifically serve for predicting the poor clinical outcome at the 3-months follow-up. We calculated the AUC regarding SAA for measuring the test accuracy. *P* < 0.05 reported statistical significance.

## 3. Results

### 3.1. Demographics and symptoms

The study included aSAH patients at the participating center from March 2016 to August 2022. We first enrolled 586 participants, followed by excluding 130 participants, including 23 participants who were not followed up at 3 months, six participants who did not provide their SAA or CRP data, and 101 participants who conformed to the exclusion criteria, such as a history of cerebrovascular disease and brain tumors, a combination of infectious diseases, immune dysfunction, hematological diseases, and severe organ function impairment, severe hepatic or renal disease, as well as refusal to undergo surgical treatment etc. Eventually, the study yielded 456 patients (208 males, aged 64.99 ± 8.54 years), that included 200 (43.86%) patients in poor outcome group after aSAH and 256 (78.29%) patients in good outcome group after aSAH. Relative to patients in good outcome group after aSAH, those in poor outcome group presented higher baseline Hunt and Hess grade (*P* < 0.001), higher modified fisher score (*P* < 0.001), higher WFNS score on admission (*P* < 0.001), higher proportions of infection (*P* = 0.009), higher WBC (*P* = 0.018), higher CRP (*P* = 0.011), higher IL-6 (*P* < 0.001), as well as higher SAA (*P <* 0.001). The basic characteristics of the 456 patients were presented in Table 1.

**Table 1:** Clinical and demographic characteristics of the samples under study.

**Variables**	**Total** **(*n* = 456)**	**3-month functional outcome**		**Healthy controls** **(*n* = 180)**	***P*** **-value**
		**Poor outcome** **(*****n*** = **200)**	**Good outcome** **(*****n*** = **256)**		
**Demographic characteristics**					
Gender, male, *n* (%)	208 (45.61)	86 (43)	122 (47.66)	90 (50)	0.373
Age, years, mean ± SD	65.96 ± 8.26	66.31 ± 7.98	65.53 ± 8.58	66.19 ± 8.43	0.556
**Vascular risk factors (%)**					
Hypertension	294 (64.47)	134 (67)	160 (62.5)		0.319
Diabetes mellitus	143 (31.36)	61 (30.5)	82 (32.03)		0.727
Current smoking	144 (31.58)	69 (34.5)	75 (29.30)		0.236
Alcohol consumption	135 (29.61)	62 (31)	73 (28.52)		0.564
**Laboratory findings (IQR)**					
WBC, × 10^9^/L, median (IQR)	6.79 (5.68–8.25)	7.15 (5.83–8.64)	6.53 (5.61–7.80)		0.004
CRP, mg/L, median (IQR)	7.56 (5.97–11.77)	10.86 (6.99–15.00)	6.45 (5.63–8.85)		< 0.001
IL−6, ng/L, median (IQR)	5.3 (4.60–8.18)	5.3 (4.9–8.2)	5.1 (4.3–7.88)		0.007
Plt, × 10^9^/L, median (IQR)	213 (174–247)	206 (173–246)	217 (176–247)		0.271
TG, mmol/L, median (IQR)	1.37 (0.96–1.99)	1.31 (0.92–2.02)	1.38 (0.97–1.93)		0.860
TC, mmol/L, median (IQR)	4.45 (3.76–5.27)	4.46 (3.76–5.05)	4.48 (3.75–5.51)		0.446
Glucose, mmol/L, median (IQR)	5.31 (4.76–6.89)	5.15 (4.70–6.70)	5.40 (4.80–6.90)		0.152
RBC, × 10^12^/L, median (IQR)	4.76 (4.51–5.22)	4.78 (4.51–5.21)	4.83 (4.52–5.25)		0.530
Hb, g/L, median (IQR)	139 (132–144)	138 (131–144)	140 (134–145)		0.098
HDL–C, mmol/L, median (IQR)	1.03 (0.86–1.24)	1.01 (0.83–1.17)	1.04 (0.86–1.30)		0.061
LDL–C, mmol/L, median (IQR)	2.52 (1.97–3.18)	2.45 (1.87–3.06)	2.56 (1.99–3.22)		0.076
ApoA, g/L, median (IQR)	1.25 (1.11–1.47)	1.26 (1.10–1.45)	1.28 (1.12–1.48)		0.362
ApoB, g/L, median (IQR)	0.87 (0.68–1.04)	0.85 (0.69–1.00)	0.87 (0.70–1.03)		0.425
Serum amyloid A, mg/L, median (IQR)	32.99 (26.53–37.44)	36.44 (32.23–41.00)^a^	28.99 (14.67–34.12)^b^	5.64 (3.43–7.45)	< 0.001
**Hunt and hess grade**, ***n*** **(%)**					
I–II	315 (69.08)	151 (75.5)	164 (64.06)		0.009
III–V	141 (30.92)	91 (45.5)	50 (19.53)		< 0.001
**Modified fisher scale**, ***n*** **(%)**					
I–II	147 (32.23)	84 (42.20)	73 (28.52)		0.042
III–IV	309 (67.76)	159 (79.5)	150 (58.59)		< 0.001
**Operation of aneurysm**, ***n*** **(%)**					0.781
Clip	87 (19.08)	37 (18.5)	50 (19.53)		
Coil	369 (80.92)	163 (81.5)	206 (80.47)		
**Aneurysm location**, ***n*** **(%)**					0.128
Anterior circulation	408 (89.47)	174 (87)	234 (91.41)		
Posterior circulation	48 (10.53)	26 (13)	22 (8.59)		
Size of aneurysm, mm, mean (SD)	4.8 (4.5–5.8)	4.8 (4.5–5.9)	4.8 (4.4–5.5)		0.448
WFNS score on admission, median (IQR)	2 (2–3)	2 (2–4)	2 (2–3)		0.002
Systolic arterial pressure, mmHg, mean (SD)	138 (130–149)	141 (130–150)	136 (130–148)		0.194
Diastolic arterial pressure, mmHg, mean (SD)	91 (89–100)	94 (90–101)	90 (89–100)		0.219
Acute hydrocephalus, *n* (%)	30 (6.58)	10 (5)	20 (7.81)		0.229
Intraventricular hemorrhage, *n* (%)	32 (7.02)	14 (7)	18 (7.03)		0.990
Seizure, *n* (%)	45 (9.87)	20 (10)	25 (9.77)		0.934
Infection, *n* (%)	144 (31.58)	76 (38)	68 (26.56)		0.009

a *P* < 0.001 compared to good outcome.

b *P* < 0.001 compared to healthy control.

### 3.2. SAA values of patients and healthy controls

**Figure 2:**
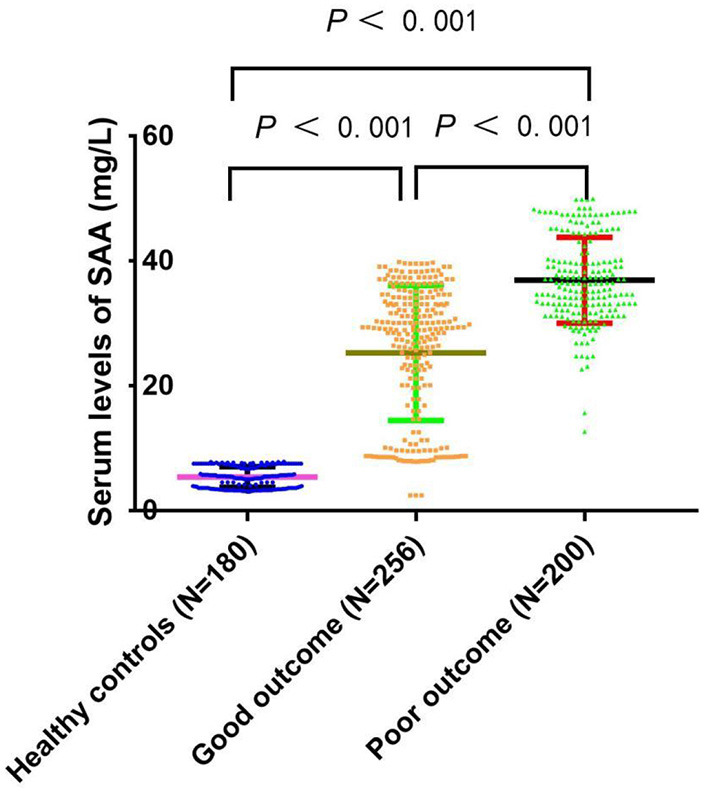
Comparison of SAA concentrations among the poor prognosis, good prognosis, and healthy controls. SAA denotes serum amyloid A. SAA concentrations are reported as the medians and interquartile ranges.

Table 1 compares the baseline characteristics between the patients and healthy controls. The median value (IQR) of SAA for all aSAH patients was significantly higher than normal subjects (32.99 (26.53–37.44) vs. 5.64 (3.43–7.45), *P* < 0.001). Moreover, a significant inter-group difference in SAA at admission was revealed (*P* < 0.001). Indeed, the results showed that the SAA of poor outcome group were significantly increased compared to good outcome group and healthy control group (36.44 (32.23–41.00) vs. 28.99 (14.67–34.12) and 5.64 (3.43–7.45), *P* < 0.001). The differences were not significant in age and sex percentage among poor outcome group, good outcome group, and healthy controls group (*P* > 0.05). Figure 2 revealed that serum SAA concentrations in patients with poor outcome were significantly higher than those in good outcome group and healthy control group.

### 3.3. Baseline characteristics exhibited by all participants in SAA tertiles

Tertiles of the SAA level were taken into account for dividing all patients into three subgroups, ensuring that each subgroup had sufficient patient categories from 2.45 to 49.98 (Q1, 152 patients; Q2, 152 patients; Q3, 152 patients). The cut-off values (COV) for stratifying the SAA into tertiles were: (Q1) 2.45–29.23, (Q2) 29.33–35.44, (Q3) 35.54–49.98. Ascending tertiles of SAA reported higher baseline Hunt and Hess grade (*P <* 0.001), higher modified fisher score (*P <* 0.001), higher WFNS score on admission (*P* = 0.015), higher WBC (*P* = 0.018), higher CRP (*P* = 0.011), and higher IL-6 (*P* < 0.001) (Table 2). The poor and good outcome groups presented significant differences in terms of the SAA (χ2 = 110.09, *P <* 0.001). Actually, for the poor outcome group, the percentage of patients in the lowest tertile (2.45–29.23) and the highest tertile (35.54–49.98) were remarkably lower and higher, respectively. Besides, there were 17 (8.5%), 77 (38.5%), and 106 (53%) poor outcome patients after aSAH in Tertile 1, Tertile 2, and Tertile 3, respectively (Table 3).

**Table 2:** Baseline characteristics of patients with aSAH according to SAA tertiles.

**Variables**	**SAA**			***P*** **-value**
	**Q1 (** ≤ **29.23**, ***n*** = **152)**	**Q2 (29.33–35.44**, ***n*** = **152)**	**Q3 (**≥**35.54**, ***n*** = **152)**	
**Demographic characteristics**				
Gender, male, *n* (%)	65 (42.76)	70 (46.05)	73 (63.16)	0.648
Age, years, mean ± SD	68 (60–71)	68 (62–71)	68 (61–71)	0.482
**Vascular risk factors (%)**				
Hypertension	90 (59.21)	100 (65.79)	104 (68.42)	0.225
Diabetes mellitus	40 (26.32)	45 (29.61)	58 (38.16)	0.071
current smoking	42 (27.63)	48 (31.58)	54 (35.53)	0.334
Alcohol consumption	40 (26.32)	50 (32.89)	45 (29.61)	0.454
**Laboratory findings (IQR)**				
WBC, × 10^9^/L, median (IQR)	6.44 (5.63–7.79)	6.7 (5.46–8.38)	7.12 (6.04–8.69)	0.016
CRP, mg/L, median (IQR)	6.63 (5.77–8.95)	8.0 (6.0–12)	8.99 (6.25–14.67)	< 0.001
IL−6, ng/L, median (IQR)	4.9 (3.9–6.4)	5.40 (4.83–8.50)	5.30 (4.80–8.50)	< 0.001
Plt, × 10^9^/L, median (IQR)	216 (161–241)	231 (173–241)	210 (180–257)	0.645
TG, mmol/L, median (IQR)	1.37 (0.99–1.93)	1.32 (0.86–1.78)	1.42 (0.72–2.26)	0.064
TC, mmol/L, median (IQR)	4.55 (3.77–5.64)	4.32 (3.74–5.05)	4.55 (3.71–5.20)	0.434
Glucose, mmol/L, median (IQR)	5.40 (4.8–6.80)	5.20 (4.70–7.20)	5.30 (4.6–6.3)	0.473
RBC, × 10^12^/L, median (IQR)	4.94 (4.56–5.25)	4.67 (4.51–5.21)	1.01 (0.82–1.17)	0.108
Hb, g/L, median (IQR)	140 (134–145)	138 (134–144)	140.0 (132.0–145.0)	0.697
HDL–C, mmol/L, median (IQR)	1.05 (0.86–1.30)	1.02 (0.86–1.27)	1.01 (0.82–1.17)	0.158
LDL–C, mmol/L, median (IQR)	2.55 (1.96–3.23)	2.46 (1.94–3.14)	2.55 (1.94–3.08)	0.448
ApoA, g/L, median (IQR)	1.29 (1.13–1.50)	1.26 (1.10–1.44)	1.27 (1.09–1.47)	0.368
ApoB, g/L, median (IQR)	0.87 (0.70–1.04)	0.86 (0.68–1.01)	0.85 (0.69–0.99)	0.630
Serum amyloid A, mg/L, median (IQR)	20.11 (8.69–26.69)	32.99 (30.86–34.12)	39.11 (37.44–44.99)	< 0.001
**Hunt & Hess grade**, ***n*** **(%)**				
I–II	84 (55.26)	96 (63.16)	135 (88.81)	< 0.001
III–V	30 (19.74)	34 (22.37)	105 (69.08)	< 0.001
**Modified Fisher scale**, ***n*** **(%)**				
I–II	37 (24.34)	45 (29.61)	65 (42.76)	0.009
III–IV	85 (55.92)	102 (67.11)	122 (80.26)	< 0.001
**Operation of aneurysm**, ***n*** **(%)**				
Clip	25 (16.45)	31 (20.39)	31 (20.39)	0.600
Coil	118 (77.63)	120 (78.95)	131 (86.18)	0.124
**Aneurysm Location**, ***n*** **(%)**				
Anterior circulation	130 (85.53)	140 (92.11)	138 (90.79)	0.141
Posterior circulation	15 (9.87)	16 (10.53)	17 (11.18)	0.933
Size of aneurysm, mm, mean (SD)	4.8 (4.5–5.7)	4.8 (4.5–5.8)	4.8 (4.5–5.8)	0.418
WFNS score on admission, median (IQR)	2 (1–3)	2 (2–3)	2 (2–3)	0.015
Systolic arterial pressure, mmHg, mean (SD)	136 (130–147)	137 (132–149)	140 (133–152)	0.058
Diastolic arterial pressure, mmHg, mean (SD)	90 (89–100)	90 (89–100)	94 (90–102)	0.108
Acute hydrocephalus, *n* (%)	9 (5.92)	10 (6.58)	11 (7.24)	0.898
Intraventricular hemorrhage, *n* (%)	10 (6.58)	11 (7.19)	11 (7.24)	0.967
Seizure, *n* (%)	14 (9.21)	18 (11.84)	13 (8.55)	0.726
Infection, *n* (%)	40 (31.58)	50 (38)	54 (26.56)	0.197

**Table 3:** SAA tertiles of patients.

**Variables**	**Poor outcome (*n* = 200)**	**Good outcome (*n* = 256)**	**χ2**	***P*** **-value**
SAA			110.09	<0.001
Tertile 1 (2.45–29.23)	17 (8.5%)	135 (52.73%)	98.86	<0.001
Tertile 2 (29.33–35.44)	77 (38.5%)	75 (29.30%)	4.28	0.039
Tertile 3 (35.44–49.98)	106 (53%)	46 (17.97%)	62.01	<0.001

SAA, serum amyloid A.

### 3.4. Association between the level of SAA and poor outcome after aSAH

We conducted the multivariate logistic regression analysis by including Hunt and Hess grade (III–IV vs. I–II), modified fisher scale (III–IV vs. I–II), WFNS score on admission, WBC, CRP, and IL-6 as independent variables, confirming that CRP (OR 1.209, 95% CI: 1.117–1.328, *P* = 0.012), SAA (OR 1.129, 95% CI: 1.081–1.177, *P* = 0.000), Hunt and Hess grade (III–IV vs. I–II) (OR 8.667, 95%CI: 7.045–12.996, *P* = 0.012), modified fisher scale (III–IV vs. I–II) (OR 6.743, 95% CI: 6.228–9.217, *P* = 0.011), and WFNS score on admission (OR 1.621, 95%CI: 1.212–2.153, *P* = 0.007) could independently predict the poor outcome at 3 months after aSAH (Table 4). Correlation analyses indicated the positive relationship between SAA and CRP (*r* = 0.297, *P <* 0.001), and the positive relationship between SAA and IL-6 in all patients on admission (*r* = 0.206, *P* = 0.024). Similarly, an obviously weak positive correlation was found between SAA and the modified fisher scores (*r* = 0.191, *P*= 0.041). Moreover, a weak positive correlation also existed between SAA and WFNS score (*r* = 0.113, *P* = 0.016).

**Table 4:** Univariate logistic regression analysis of 3-month poor outcome after aSAH.

**Variables**	**OR**	**95% CI**	***P*** **-value**
Infection	1.106	0.859–1.332	0.286
WBC	1.062	0.912–1.256	0.349
CRP	1.209	1.117–1.328	0.012
IL−6	1.072	0.867–1.131	0.081
SAA	1.129	1.081–1.177	< 0.001
Hunt and Hess grade (III–IV vs. I–II)	8.667	7.045–12.996	0.012
Modified Fisher Scale (III–IV vs. I–II)	6.743	6.228–9.217	0.011
WFNS score on admission	1.621	1.212–2.153	0.007

**Figure 3:**
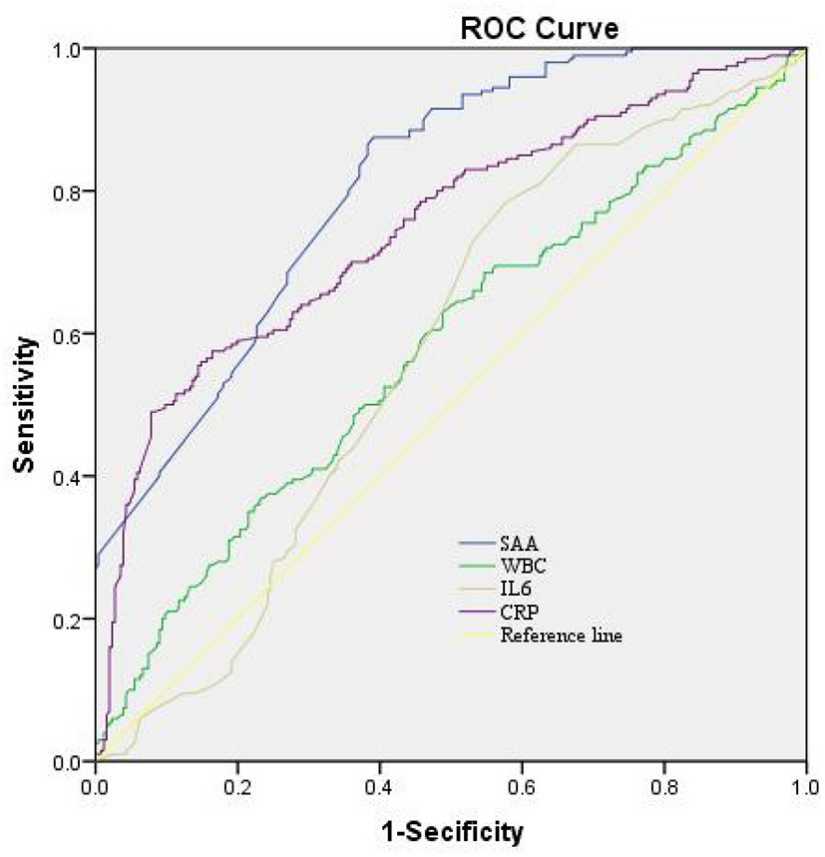
The ROC curves for the prediction of poor outcome. Predictive values of SAA, CRP, IL-6, and WBC for depression at 3-month after aSAH. AUC 0.573 (95%CI, 0.521–0.626; *P* = 0.004) for IL-6; 0.748 (95%CI, 0.702–0.794; *P* = 0.007) for CRP; 0.579 (95%CI, 0.526–0.632; *P* = 0.004) for WBC; and 0.807 (95%CI, 0.623–0.747; *P* = 0.004) for SAA. SAA had the COV of 30.28, and the sensitivity and specificity were 61.9 and 78.7%, respectively. ROC, receiver operating characteristic; SAA, serum amyloid A; AUC, area under the curve; CI, confidence interval; CRP, C-reactive protein; IL-6, interleukin-6; WBC, White blood cell; COV, cut-off value.

In the logistic regression model without making any adjustments and the model with multiple adjustments, we took all participants as a whole, considering the poor outcome and the lowest tertile as the dependent variable and the reference, respectively for investigating SAA (Table 5). In the logistic regression model without making any adjustments, for patients with admission SAA, the highest quartile presented more poor outcome relative to the lowest tertile (non-adjusted: OR 2.780, 95% CI: 1.224–6.313, *P* < 0.001). In the logistic regression model that adjusted for the confounders of age, sex, vascular risk factors (hypertension, diabetes mellitus, current smoking, alcohol consumption), hunt and hess grade (III–V vs. I–II), modified fisher scale (III–IV vs. I–II), and WFNS score on admission, as well as laboratory data (WBC, CRP, and IL-6), the highest tertile of SAA could independently predict poor outcome prevalence (model 1B: OR = 2.362, 95% CI = 1.139–4.896, *P* = 0.021; model 2C: OR = 2.259, 95% CI = 1.070–4.770, *P* < 0.001; model 3D: OR = 2.247, 95% CI =1.095–4.604, *P* = 0.021). As revealed by the ROC curve, the estimated optimal cutoff of SAA that predicted the poor outcome was 30.28, with the sensitivity and specificity reaching 61.9 and 78.7%, respectively and the AUC at 0.807 (95%CI, 0.769–0.846; *P* = 0.004) (Figure 3).

**Table 5:** Unadjusted and adjusted associations between quartile of SAA levels and poor outcome at 90 days.

	**Tertile**	**OR^A^**	**95% CI**	***P*** **-value**
Unadjusted	Middle	2.601	0.757–8.123	0.026
	Highest	2.780	1.224–6.313	<0.001
Model 1B	Middle	2.162	0.636–7.350	0.217
	Highest	2.362	1.139–4.896	0.021
Model 2C	Middle	2.102	0.623–7.496	0.224
	Highest	2.259	1.070–4.770	0.033
Model 3D	Middle	2.144	1.139–3.867	0.124
	Highest	2.247	1.095–4.604	0.021

A Reference OR (1.000) is the lowest tertile of SAA for poor outcome after aneurysmal subarachnoid hemorrhage.

B Model 1: adjusted for age, sex.

C Model 2: adjusted for covariates from Model 1 and further adjusted for Vascular risk factors (Hypertension, diabetes mellitus, current smoking, alcohol consumption).

D Model 3: adjusted for covariates from Model 2 and further adjusted for variables with *p* < 0.05 in univariate analysis (WBC, CRP, IL−6, infection, hunt and hess grade (III–V vs. I–II), modified fisher scale (III–IV vs. I–II), and WFNS score on admission).

## 4. Discussion

To our best knowledge, the study is the first prospective cohort study exploring the relationship between SAA and poor prognosis at 3 months in aSAH patients. The main findings in the current study were that, (1) serum concentrations of SAA were significantly higher in patients with poor outcome of aSAH than in patients with favorable outcome of aSAH; (2) Serum SAA concentrations in aSAH patients were positively correlated with admission WFNS score, Modified Fisher score, IL-6 concentrations and serum CRP concentrations; (3) serum SAA emerged as an independent predictor for 3-month worse prognosis; (4) compared to CRP and IL-6, serum SAA concentrations were highly discriminatory for clinical outcomes under the ROC curve; (5) high-level SAA at admission exhibited an obvious relation to the poor outcome 3 months after aSAH and documented that after adjusting major confounders, patients in the highest SAA quartile presented a poor outcome risk 2.247-fold higher than those in the lowest SAA quartile, which epidemiologically proved the potential effectiveness of SAA for predicting the poor outcome at 3 months. These findings suggest that increased serum SAA concentrations may be highly correlated with inflammation and closely associated with a worsening prognosis of aSAH. Therefore, SAA may be a useful therapeutic target for the treatment of patients with aSAH. Here, SAA can well serve for treating aSAH patients as an useful therapeutic target.

Inflammation has been well-documented to be implicated in the pathogenesis of brain injury associated with aSAH. The balance between inflammatory and anti-inflammatory factors clearly affects the development of aSAH (5–7). SAA is an acute phase response protein produced by hepatocytes and belongs to a highly heterogeneous class of proteins in the apolipoprotein family. SAA is the main becoming amyloid A protein serum precursor in amyloidosis, which is composed of the same cluster of genes encoding a polymorphic protein that is more common in various inflammatory responses (18). SAA exerts immunomodulatory effects and assists in tissue regeneration by activating collagenases and acts as a chemotactic agent for monocytes, T cells, mast cells and neutrophils (19). SAA concentrations are elevated to varying degrees in various infectious and inflammatory diseases as well as in oncological diseases (18). Recently, accumulating evidence has shown that SAA plays a crucial role in the inflammation of several human diseases, e.g., acute primary basal ganglia hemorrhage, ischemic stroke, inflammatory rheumatic diseases, acute myocardial infarction, acute myocardial infarction, traumatic brain injury, and hypoxic ischemic encephalopathy (13, 20–24). The current study verified a similar result that there was a significant elevation of serum SAA concentrations after aSAH, as compared to normal reference values. In addition, a significantly higher concentration of SAA has been reported in mice with cerebral ischemia, and it has also been found to mediate microglia activation *via* gene knockout techniques (25). Alternatively, a recent animal study on mice with traumatic brain injury revealed that serum SAA may be a novel neuroinflammation-based, and severity-dependent, biomarker for acute traumatic brain injury (26). Recent studies have found that elevated IL-6 levels induce neuroinflammation and may be strongly associated with poor outcomes of aSAH (27). CRP, similar to SAA, is an acute-phase protein whose peripheral blood concentration not only reflects the degree of systemic inflammatory response, but also the degree of inflammatory response in the brain (28–30). The interesting finding in this study was that serum SAA concentrations were in close correlation with systemic inflammation reflected by CRP and IL-6 concentrations. Therefore, it is assumed that serum SAA might at least be involved in the acute systemic inflammation caused by aSAH brain injury.

Up until now, it remains unclear whether circulating SAA is associated with the prognosis of acute brain injury. In the present study of 456 patients with aSAH, we found that serum SAA concentrations were strongly correlated with WFNS scores as well as modified Fisher scores. Consistent with previous studies on acute cerebral hemorrhage (13), on the other hand, we support the hypothesis that serum SAA concentrations can reflect the clinical severity after acute brain injury, including acute cerebral hemorrhage and aSAH. Our study revealed that serum SAA was independently associated with 90-day poor outcome even after correcting for conventional prognostic determinants, such as WFNS scores and modified Fisher scores, which are the two common determinants for prognosis of aSAH (8, 31–33). In addition, under ROC curve, serum SAA concentrations showed significant prognostic accuracy in differentiating patients with poor prognosis from those with good prognosis at 3 months after aSAH. Previous extensive numerous studies had revealed that patients with a poor prognosis after aSAH had a significant increase in inflammatory or inflammatory-related disease markers including IL-33, serum stanniocalcin 1, and red blood cell distribution width (8, 32, 34). Interestingly, we performed some discriminatory ability comparisons of AUC between serum SAA concentrations and three common biochemical variables, namely serum CRP concentrations, serum IL-6 concentrations, and blood leukocyte counts (35–37). It was demonstrated that the prognostic predictive ability of serum SAA concentrations were considerably higher than that of serum CRP concentrations, IL-6 concentrations, and blood leukocyte count. At the same time, the optimal point of serum SAA concentration was selected for the differentiation of those patients with poor prognosis with moderate-high sensitivity and specificity. In other words, serum SAA could be used as a promising prognostic biomarker for the treatment of human aSAH.

## 5. Conclusions

The current study 456 aSAH patients have demonstrated that increased SAA concentrations in circulating blood have relation to admission WFNS scores and modified Fisher scores in addition to peripheral CRP concentrations, as well as serum SAA concentrations are independently associated with poor outcome at 3 months after aSAH. An intriguing finding of this study was the high discriminatory ability of serum SAA concentrations for poor outcomes after aSAH. In summary, it is demonstrated that that SAA on admission may be an important biomarker of inflammatory disease assessing the prognosis after aSAH.

## Data availability statement

The raw data supporting the conclusions of this article will be made available by the authors, without undue reservation.

## Ethics statement

The studies involving human participants were reviewed and approved by the First Affiliated Hospital of Anhui University of Science and Technology (First People's Hospital of Huainan). Participants provided informed consent prior to inclusion in this study.

## Author contributions

ZS and YL designed the research study and analyzed the data. YL, FC, KJ, and ZS performed the research. YL wrote the manuscript. All authors contributed to editorial changes in the manuscript and read and approved the final manuscript.
